# Investigate the Odontogenic Differentiation and Dentin–Pulp Tissue Regeneration Potential of Neural Crest Cells

**DOI:** 10.3389/fbioe.2020.00475

**Published:** 2020-06-05

**Authors:** Maolin Zhang, Xiaochen Zhang, Jiaxin Luo, Ran Yan, Kunimichi Niibe, Hiroshi Egusa, Zhiyuan Zhang, Ming Xie, Xinquan Jiang

**Affiliations:** ^1^Department of Prosthodontics, School of Medicine, Ninth People's Hospital Affiliated to Shanghai Jiao Tong University, Shanghai, China; ^2^Shanghai Key Laboratory of Stomatology & Shanghai Research Institute of Stomatology, National Clinical Research Center of Stomatology, Shanghai, China; ^3^Division of Molecular and Regenerative Prosthodontics, Tohoku University Graduate School of Dentistry, Sendai, Japan; ^4^Department of Oral and Maxillofacial-Head and Neck Oncology, School of Medicine, Ninth People's Hospital, Shanghai Jiao Tong University, Shanghai, China

**Keywords:** neural crest cells, iPSC-derived neural crest-like cells, odontogenic differentiation, dentin–pulp complex regeneration, tooth regeneration

## Abstract

Stem cell-based developmental engineering has been considered as a promising strategy for tissue/organ regeneration. Tooth is formed by sequential reciprocal interactions between epithelium derived from surface ectoderm and mesenchymal cells derived from cranial neural crest. The neural crest cell is an appealing cell source for tooth development and regeneration research. In this study, we investigated the odontogenic differentiation and dentin-pulp complex regeneration potential of neural crest cells. Our results showed that neural crest cells (O9-1 mouse cranial neural crest cell line) can sequentially differentiate into dentin matrix acidic phosphoprotein 1 (DMP-1)-positive odontoblasts within a developing tooth germ *in vitro*. Moreover, O9-1 cells and induced pluripotent stem cell (iPSC)-derived neural crest-like cells (iNCLCs) can form well-organized vascularized dentin-pulp complex when transplanted *in vivo* with tooth scaffold. Furthermore, both O9-1 cells and iNCLCs can be differentiated into odontoblast-like cells, positive staining with odontogenic-related markers DMP-1 and dentin sialophosphoprotein (DSPP), under odontogenic induction with the administration of bone morphogenetic protein 4 (BMP-4). These results demonstrated that neural crest cells, especially the unlimited iNCLCs, are a promising cell source for tooth development and dental tissue/tooth organ regeneration studies.

## Introduction

Tooth damage or loss, caused by dental carious, periodontitis, or traumatic injury, has a severe impact on our life. Restoration of missing teeth is difficult at present without using artificial materials. Tooth replacement via regenerative therapy is thought to be an attractive new concept in contemporary dentistry or stomatology. The main concept in current tooth regeneration is following the cues of natural tooth development process either *in vitro* or *in vivo* using stem cells. During embryonic development, tooth is formed by sequential reciprocal interactions between epithelium derived from surface ectoderm (Biggs and Mikkola, 2014) and mesenchymal cells derived from cranial neural crest (Kollar and Fisher, 1980; Chai et al., 2000). The cranial neural crest cells migrate to pharyngeal arches and contribute to a broad variety of derivatives, including craniofacial bone, cartilage, connective tissue, and teeth (Santagati and Rijli, 2003; Noden and Trainor, 2005; Kulesa et al., 2010). Since the pluripotent differentiation potential of neural crest cells (NCCs), they have been widely investigated in cell-based tissue regeneration and disease-specific repair (Achilleos and Trainor, 2012). Thus, NCCs is an ideal candidate for the study of tooth development and regeneration *in vitro* and *in vivo* (Xing et al., 2016). However, neural crest is a temporary embryonic structure in vertebrates. Even though there were reports that neural crest stem cells still present in the adult tissues such as gingiva (Zhang Q. et al., 2018), bone marrow (Morikawa et al., 2009; Niibe et al., 2017), and dental periodontal tissues (Ibarretxe et al., 2012), it is quite difficult to isolate plenty of primary NCCs for the research of stem cell-based tooth development and regeneration.

Induced pluripotent stem cells (iPSCs), reprogrammed from somatic cells via genetic modification, possess embryonic stem cell (ESCs) characteristics (Takahashi and Yamanaka, 2006; Takahashi et al., 2007) and have been considered as promising cell sources for regenerative medicine (Xu et al., 2014). Previous studies have demonstrated that NCCs can be isolated from pluripotent stem cells including ESCs and iPSCs (Lee et al., 2007; Liu et al., 2012). Moreover, iPSC-derived neural crest like cells (iNCLCs) can further differentiate into odontogenic cells by administration of recombinant growth factors, such as bone morphogenetic protein 4 (BMP-4) and fibroblast growth factor 8 (FGF-8) (Kawai et al., 2014; Kidwai et al., 2014), or by gene transfection (Seki et al., 2015), or by direct or indirect coculture with odontogenic cells (Otsu et al., 2012; Seki et al., 2015). However, there are very rare reports about direct observation of how NCCs sequentially differentiate into an odontoblast within a developing tooth germ *in vitro* or form well-organized dental tissue *in vivo*.

In this study, we investigated the odontogenic differentiation and dental tissue formation potential of NCCs. To the best of our knowledge, this is the first report of the direct observation of the sequential differentiation of NCCs to a DMP-1-positive odontoblast within the developing tooth germ *in vitro*. Moreover, both primary O9-1 mouse cranial neural crest cell line and iNCLCs can contribute to well-organized dentin-pulp complex regeneration *in vivo* and differentiate into odontoblast-like cells *in vitro*. These results demonstrated that NCCs, especially unlimited iNCLCs, are promising cell sources for the research related to tooth development and regeneration.

## Materials and Methods

### Cell Culture

Mouse gingiva-derived iPSCs were maintained on mitomycin-C-treated mouse embryonic fibroblasts (MEF, Gibco) feeder layers on gelatin-coated dishes in Dulbecco's modified Eagle's medium (DMEM, Invitrogen) supplemented with 15% fetal bovine serum (FBS, Gibco), 2 mM l-glutamine (Gibco), 1 × 10^−4^ M non-essential amino acids (Stemcell Technologies), 1 × 10^−4^ M 2-mercaptoethanol (Sigma), 50 U penicillin, and 50 μg/ml streptomycin) (Okawa et al., 2016).

O9-1 mouse cranial neural crest cell line was purchased from EMD Millipore (Millipore, Temecula, CA). The cells were cultured in complete embryonic stem (ES) cell medium (Millipore, Temecula, CA) supplemented with 25 ng/ml FGF-2 (Millipore, Temecula, CA).

### Recombination of Epithelial and Mesenchymal Cells

To reconstitute a tooth germ *in vitro*, molar tooth germs were dissected from the mandibles of ED14.5 C57BL/6J mice (Ikeda et al., 2009). The isolated tissues were treated by 50 U/ml Dispase I (BD, Franklin Lakes, NJ) for 10 min, and then, dental epithelium and dental mesenchyme were mechanically separated using a 25-G needle. To directly investigate the potential contribution to bioengineered tooth formation of different cell populations including O9-1 cells, human dental pulp stem cells (hDPSCs), and NIH3T3 (all three cell populations were labeled with green fluorescent protein (GFP) lentivirus; hDPSCs and NIH3T3 were treated as control), the cells were mixed with the single dental mesenchymal cells derived from dental mesenchyme at different ratio (Table S1) as previously described (Yang et al., 2017). The mixed cell pellets (total 3 × 10^5^) were recombined with one piece of dental epithelial tissue in a 30 μl gel drop (Cellmatrix Type I-A, Nitta Gelatin) and placed on a cell culture insert (8.0 μm pore size; 353182, BD) (Figure S1A). The reconstituted complexes were cultured up to 14 days with DMEM supplemented with 10% FBS, 100 μg/ml ascorbic acid (Sigma), and 2 mM l-glutamine (Sigma).

To distinguish the epithelium and mesenchyme and further trace the biological behavior of O9-1 cells in the developing tooth germ, the dental epithelial tissue was isolated from Rosa26 (ACTB-tdTomato,-EGFP) mice that express strong red fluorescence in all tissues, and O9-1 cells were labeled with GFP lentivirus. The morphology of the reconstituted complex was observed at days 1, 4, 7, 11, and 14 using a fluorescence microscope (OLYMPUS, Tokyo, Japan).

For tissue immunofluorescent staining, tissue samples were embedded in Tissue-Tek OCT (Sakura Finetek, USA). Serial frozen sections were cut at a thickness of 6 μm; the sections were incubated with the primary antibody against DMP-1 (sc-73633, 1:100, Santa Cruz Biotechnology Inc.) overnight at 4°C. Then, the sections were washed with phosphate-buffered saline (PBS) and incubated with Alexa 594-labeled second antibody for 1 h at room temperature. After rinsing with PBS for three times, the sections were incubated with 4′6-dia-mino-2-phenylindole (DAPI) (Wako, Osaka, Japan) for 10 min at room temperature. These sections were observed under a fluorescence microscope.

### iNCLCs Induction

iPSCs were harvested using 0.25% trypsin and seeded in six-well low-cell-adhesion plates at a density of 3 × 10^5^ cells/well with neural induction medium following previous studies to form neural spheres (Otsu et al., 2012). The spheres were then transferred to a fibronectin-coated dish and cultured for 1 week in neural induction medium. The remaining spheres were mechanically removed, and the outgrowth iNCLCs were passaged using 0.25% trypsin and cultured on fibronectin-coated dish (Otsu et al., 2012). The expression of neural-crest-related markers including AP-2, p-75, and Nestin were evaluated using conventional reverse transcription PCR (RT-PCR) and immunohistochemistry analysis. The corresponding primer sequences are displayed in Table 1.

**Table 1:** Nucleotide sequences for reverse transcription PCR (RT-PCR) primers.

**Gene**	**Primer sequence (5^**′**^-3^**′**^) (forward/reverse)**	**Product size (bp)**	**Accession number**
AP2	ACCAGCAACGGGACGGCAAGG	517	NM_001122948.2
	TGGCGGAGACAGCATTGCTGTTG		
p75	GGCTACTACCAGGACGAGGAG	446	NM_033217.3
	GCCAAGATGGAGCAATAGACA		
Nestin	AATGGGAGGATGGAGAATGGAC	492	NM_016701.3
	TAGACAGGCAGGGCTAGCAAG		
GAPDH	CACCATGGAGAAGGCCGGGG	418	NM_001289726.1
	GACGGACACATTGGGGGTAG		
Msx-1	CAGAAGATGCTCTGGTGAAGGC	138	NM_010835.2
	GGTTGGTCTTGTGCTTGCGTAG		
Runx-2	CGGGCTACCTGCCATCAC	78	NM 001146038.2
	GGCCAGAGGCAGAAGTCAGA		
DMP-1	CAGTGAGGATGAGGCAGACA	175	NM_001359013.1
	TCGATCGCTCCTGGTACTCT		
Dspp	AACTCTGTGGCTGTGCCTCT	171	NM_010080.3
	TATTGACTCGGAGCCATTCC		
GAPDH	TGCACCACCAACTGCTTAG	177	NM 001289726.1
	GGATGCAGGGATGATGTTC		

The multipotent differentiation potential of iNCLCs was investigated as previous described (Ishii et al., 2012). For osteogenic differentiation, the iNCLCs were cultured in osteogenic medium (DMEM, 10% FBS, 1% penicillin/streptomycin, 50 μg/ml l-ascorbic acid, 10 mM glycerophosphate and 100 nM dexamethasone, and 100 ng/ml BMP-2) for 4 weeks. The cells were fixed using 95% ethanol and stained with 1% Alizarin Red S (Sigma) for 30 min at room temperature. For adipogenic differentiation, the iNCLCs were cultured in adipogenic medium containing 0.5 mM isobutylmethylxanthine (Sigma), 0.5 mM hydrocortisone, and 60 mM indomethacin (Sigma), which was replaced every 3 days for 4 weeks. The cells were fixed using 4% paraformaldehyde and stained with Oil Red O in isopropanol for 30 min at room temperature. For chondrogenic differentiation, the monolayer culture was initially treated with the osteogenic medium for 3 days. Then, cells were cultured as a micromass format in a chondrogenic medium (Cyagen, China) containing 10% FBS, 1% penicillin/streptomycin, 1% insulin-transferrin-sodium selenite (ITS) (Sigma), 0.1 mM l-ascorbate-2-phosphate, 0.4 mM proline (Sigma), 100 nM dexamethasone, 10 ng/ml transforming growth factor-β3 (TGFβ3, Sigma), and 10 ng/ml BMP-2 for 4 weeks. Chondrogenic differentiation was detected by Safranin O staining.

### Immunocytochemistry

For immunocytochemistry analysis, the cells were incubated with anti-AP2a (ab108311, 1:300, Abcam, Cambridge), anti-p75 NGF receptor (GTX102262, 1:200, GeneTex, San Antonio, TX), DSPP (sc-73632, 1:100, Santa Cruz Biotechnology Inc.), and DMP-1 (sc-73633, 1:100, Santa Cruz Biotechnology Inc.) overnight at 4°C, respectively. Then, the cells were incubated with corresponding second antibody labeled by Alexa Fluor®594 or Alexa Fluor®488, and cell nuclei were counterstained with DAPI. The immunofluorescent images were obtained by fluorescence microscopy.

### Flow Cytometry

The iPSCs and iNCLCs were incubated with anti-p75 NGF receptor (GTX102262, 1:200, GeneTex, San Antonio, TX) at 4°C for 30 min, and then, the cells of each group were washed three times with cold PBS and incubated with fluorescein isothiocyanate (FITC)-conjugate secondary antibody. Cells were analyzed with a flow cytometer to calculate the percentage of p-75-positive cells. Experiments were performed in triplicate.

### Preparation of Human Tooth Scaffolds

Healthy human premolars and molars were collected after tooth extractions. All procedures were approved by the Ethics Committee of Shanghai Ninth People's Hospital Affiliated to Shanghai Jiao Tong University School of Medicine. The teeth were dissected around their cervical part with a dental bur at a thickness of 2 mm. The tooth scaffolds were decellularized and decalcified in ethylene diamine tetraacetic acid (EDTA), followed by copious PBS washing (He et al., 2019). The decellularized tooth scaffolds were sterilized with ethylene oxide for *in vivo* transplantation.

### Subcutaneous Transplantation

O9-1 cells and iNCLCs were separately collected and resuspended at a final concentration (2 × 10^7^ cells/ml). The cell suspension was mixed with Matrigel (BD Biosciences, Bedford, MA) at 1:1 ratio, and then, the mixture was seeded into the chamber of the tooth scaffold. Scaffold/cells complex were incubated for 15 min at 37°C to allow solidification of the Matrigel. Then, the scaffold/cell complex was subcutaneously transplanted into 6 week-old athymic nude mice. All animal experiments conducted in this study were approved by the Animal Research Committee of the Ninth People's Hospital, Shanghai Jiao Tong University School of Medicine.

### Histological and Immunohistochemical Analysis

The transplants were extracted 8 weeks after operation, fixed with 10% formaldehyde solution, decalcified with ethylene diamine tetraacetic acid (EDTA), and embedded in paraffin. A series of 5 μm sections were cut, and the sections were stained with hematoxylin-eosin (HE) for histological analysis. Immunohistochemistry was performed to analyze the newly formed tissue. The sections were incubated with primary antibodies against DSPP (sc-73632, 1:100, Santa Cruz Biotechnology Inc.), GFP (1:100, Abcam), and CD31 (1:100, Abcam) overnight at 4°C. The slides were then incubated with horseradish peroxidase (HRP)-conjugated secondary antibody, a diaminobenzidine (DAB) kit (Sigma) was used to stain the slides, and the sections were counterstained with hematoxylin. For DSPP staining, mouse femur bone tissue was treated as negative control. The number of blood vessels within the newly formed dental-pulp-like tissue was calculated using the average value of the three parallel slices (40× magnification) selected from each of the three equally divided paraffin parts along the cross-section.

### Odontoblastic Differentiation *in vitro*

For odontoblastic differentiation, the O9-1 cells and iNCLCs were cultured on collagen I (Sigma)-coated dishes. Cells were incubated with odontogenic induction medium-DMEM (Invitrogen) supplemented with 100 nM dexamethasone (Sigma), 5 mM β-glycerophosphate (Sigma-Aldrich), 50 μg/ml ascorbate phosphate (Sigma-Aldrich), 10% FBS (Invitrogen), and 100 ng/ml BMP-4 (R&D). The expression of odontogenic-related gene including *Msx-1, Runx-2, Dmp-1*, and *Dspp* were evaluated using real-time RT-PCR on day 10. The corresponding primer sequences are displayed in Table 1. Alkaline phosphatase (ALP) staining was performed using an ALP kit (Beyotime, China) on day 10 (Zhang M. et al., 2018).

### Statistical Analysis

In this study, the experimental data are presented as the mean ± standard derivation (SD). Statistical analysis was performed using one-way ANOVA with Tukey's multiple comparison test to evaluate the difference among multiple experimental groups. *P* < 0.05 was considered statistically significant.

## Results

### O9-1 Cells Contribute to Tooth Germ Formation *in vitro*

To investigate the contribution of different mesenchymal cell population to bioengineered tooth germ formation, the mixtures of 25, 50, and 100% of O9-1 cells, hDPSCs, or NIH3T3 with E14.5 dental mesenchymal cells were combined with E14.5 dental epithelium (Figure S1A). Tooth germ formation was observed only in the mixtures of 25% of NIH3T3 group and in the mixtures of 25 and 50% of O9-1 cell groups (Table S1). There were no GFP-labeled NIH3T3 cells observed inside the tooth germ (Figure S1B). NIH3T3 cells did not contribute to tooth germ formation (Figure S1B). No tooth germ formation was observed in the mixtures of any percent of hDPSCs (Table S1).

**Figure 1:**
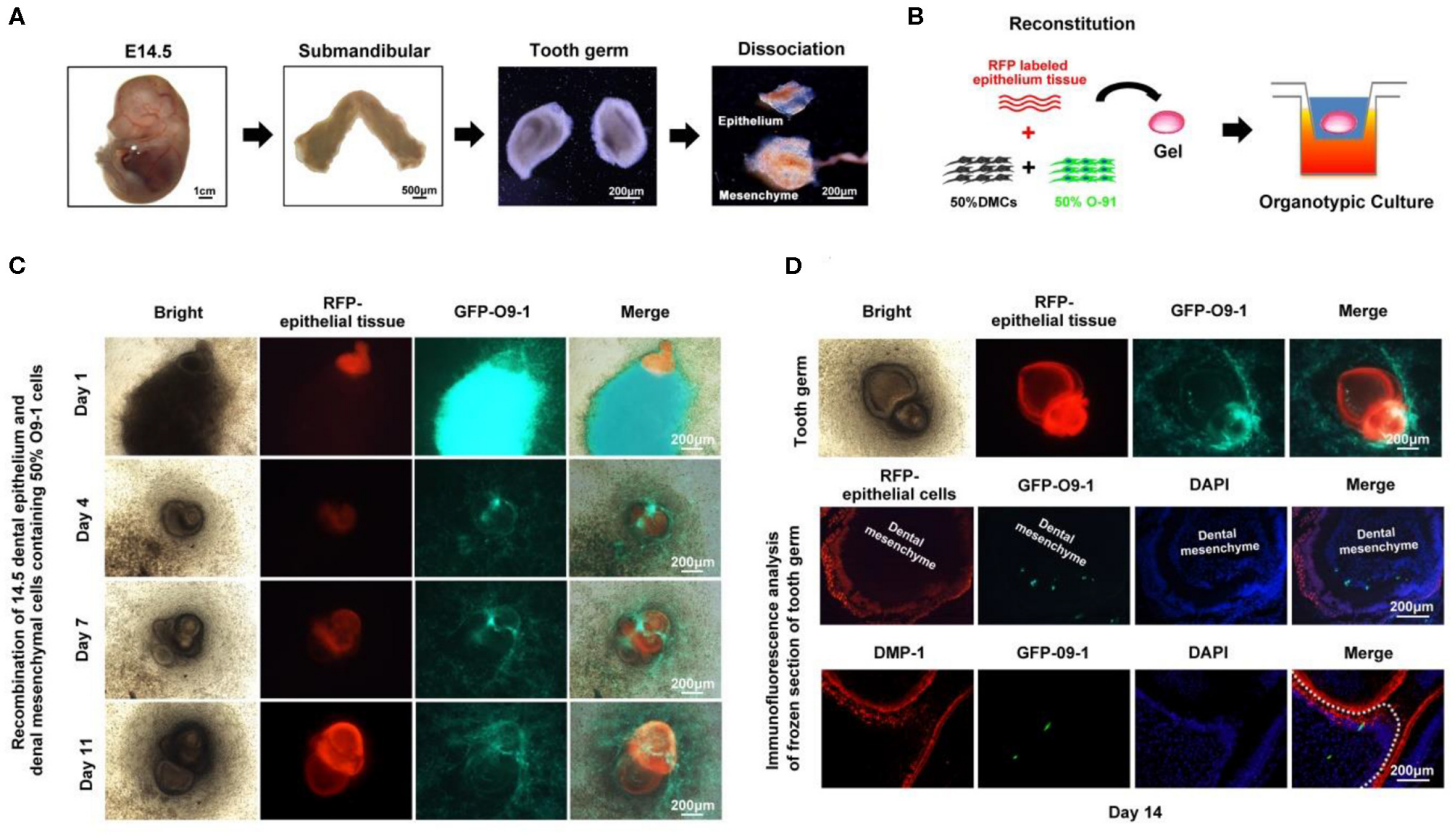
O9-1 cells contribute to tooth germ formation *in vitro*. **(A)** Tooth germs were isolated from submandibular bone at embryonic day 14.5, and the epithelial and mesenchymal tissues were microdissected. **(B)** Schematic of tooth germ reconstitution and culture *in vitro*. The dental epithelial tissue isolated from molar tooth germ of ED 14.5 Rosa26 (ACTB-tdTomato,-EGFP) mice that express strong red fluorescence. The dental mesenchyme was isolated from the molar tooth germ of ED 14.5 C57BL/6 mice. The dental mesenchyme were completely dissociated into single cells and mixed with GFP-labeled O9-1 cells at 1:1 ratio. **(C)** Time course images of the reconstitute tooth germ under organ culture *in vitro*. Red area represents dental epithelium; green area represents the mixture of O9-1 cells and dental mesenchymal cells. **(D)** The images of reconstituted tooth germ of day 14 and immunohistochemical analysis of the reconstituted tooth germ of day 14. The frozen sections of the reconstituted tooth germ were stained with DMP-1, dental mesenchyme below dash white line. Nuclei were stained with 4′,6-diamidino-2-phenylindole (DAPI).

To further test the contribution of O9-1 cells to bioengineered tooth germ formation, we utilized genetically marked mouse line, in which the dental epithelium was labeled with red fluorescence. The tooth germs were isolated from submandibular bone at embryonic day 14.5, and then, the epithelial and mesenchymal tissues were microdissected (Figure 1A). The dental mesenchyme was completely dissociated into single cells and mixed with O9-1 cells at 1:1 ratio (Figure 1B). The mixed cell pellets were recombined with the dental epithelial tissue for organ culture (Figure 1B). To distinguish the epithelium and mesenchyme and trace the biological behavior of O9-1 cells in the developing tooth germ, the dental epithelial tissue isolated from Rosa26 mice showed strong red fluorescence, and the O9-1 cells labeled by GFP showed strong green fluorescence in the reconstituted complex (Figure 1C). Following tooth organ culture, we observed the formation of tooth germ formed by the reconstituted dental epithelial and mixed dental mesenchymal cells and O9-1 cells (Figures 1C,D). The epithelial tissue showed strong red fluorescence in the tooth germ. Part of the GFP-positive O9-1 cells integrated into the developing tooth germ (Figures 1C,D). The frozen sections of the tooth germ revealed that part of the GFP-labeled O9-1 cells participated in tooth germ formation (Figure 1D). The GFP-positive cells, which were adjacent to the red fluorescence positive inner dental epithelium, exhibited an elongated appearance resembling odontoblast and showed positive staining with the odontoblastic-related marker DMP-1 (Figure 1D). These results demonstrated that O9-1 cells (mouse cranial neural crest cell line) can differentiate into odontoblasts and contribute to tooth germ formation.

### O9-1 Cells Contribute to Vascularized Dentin-Pulp Complex Regeneration *in vivo*

**Figure 2:**
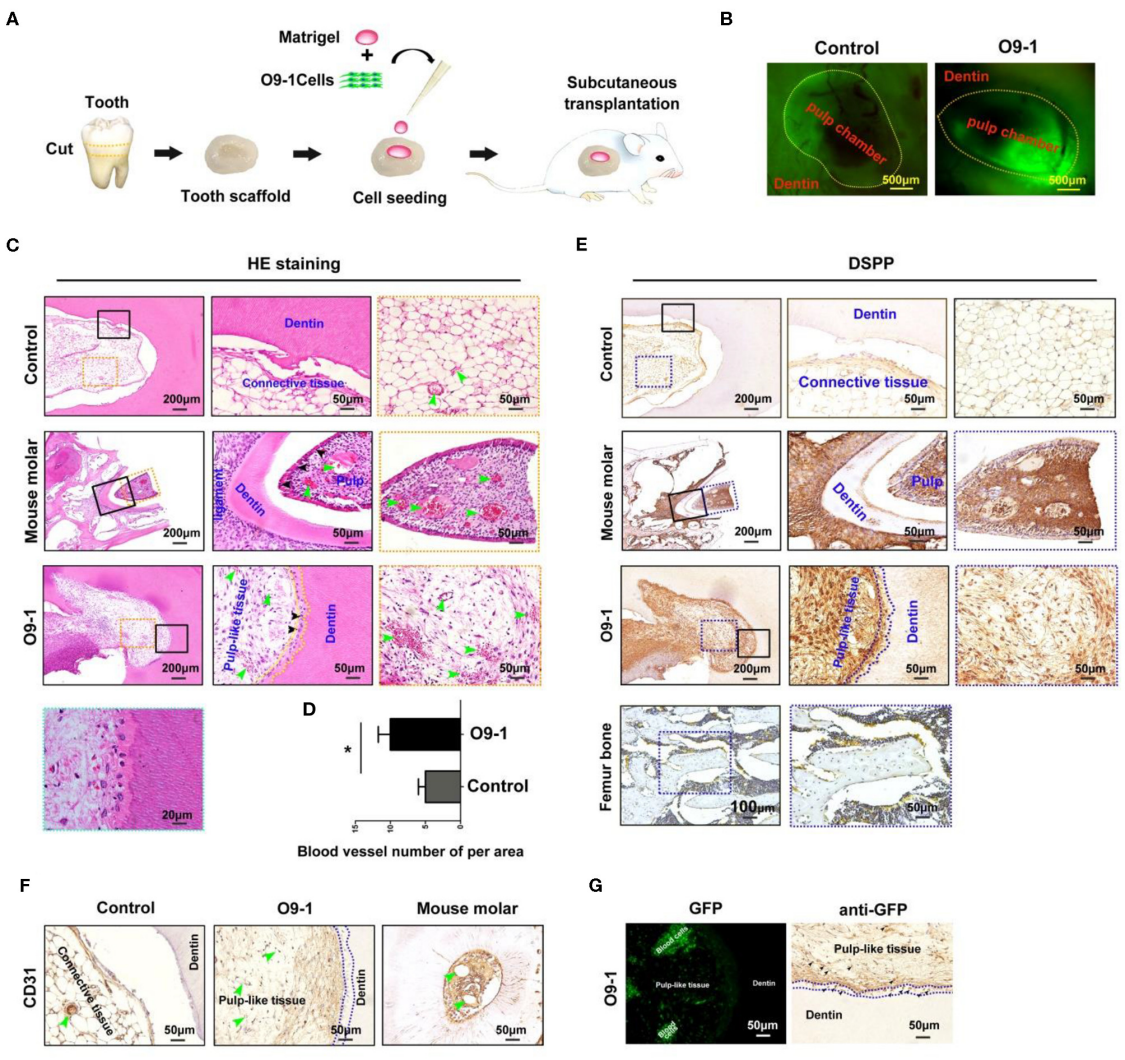
O9-1 cells contribute to vascularized dentin–pulp complex regeneration *in vivo*. **(A)** Schematic representation of the protocol for investigating the potential of O9-1 cells for pulp–dentin complex regeneration *in vivo*. **(B)** The implants were harvested after 8 weeks transplantation, and the green fluorescent protein (GFP) was observed using a fluorescence microscope. **(C)** HE staining images of each group (the right two panels are the magnification of the boxed region; black arrowheads indicate odontoblast-like cells, yellow dash line area indicates newly formed dentin, and green arrowheads indicate blood vessel). **(D)** Histomorphometric analysis of newly formed vessel area; **(E)** Dentin sialophosphoprotein (DSPP) immunohistochemical staining of each group (the right two panels are the magnification of the boxed region; dash line area indicates newly formed dentin). Femur bone tissue was treated as the negative control group. **(F)** CD31 immunohistochemical staining of each group (greenheads arrow indicate blood vessel; blue dash line area indicates newly formed dentin). **(G)** GFP expression in pulp-like tissue was observed using a fluorescence microscope and evaluated using immunohistochemistry (blue dash line area indicates newly formed dentin; black arrowheads indicate GFP-positive cells). *P < 0.05.

GFP-labeled O9-1 cells were mixed with Matrigel and seeded into the chamber of tooth scaffold, and then, the complexes were subcutaneously transplanted into the dorsum of nude mice for 8 weeks as shown in Figure 2A. The implants were extracted at 8 weeks after operation. The chamber area showed positive GFP fluorescence in O9-1 group. This demonstrated that the transplanted O9-1 cells survive well *in vivo* (Figure 2B). Histological analysis revealed that O9-1 cells formed apparent dentin–pulp complex within the tooth scaffold chamber (Figure 2C, *n* = 4, total six implants). Odontoblast-like cells, resembling the odontoblasts of mouse molar, lined the surface of the newly formed dentin; the newly formed dentin was surrounded by pulp-like connective tissue, infiltrating with blood vessels (Figure 2C). There was no dentin-pulp complex formation in the control group. Only loose connective tissue was observed in the tooth scaffold chamber (Figure 2C). The number of newly formed blood vessels within the pulp-like tissue was higher than within the loose connective tissue of the control group (Figure 2D). In addition, the newly formed pulp-like tissue and odontoblast-like cells by O9-1 cells, similar with dental pulp tissue of mouse molar, showed positive staining with DSPP (Figure 2E). The mouse femur bone tissue was regarded as negative control. The expression of DSPP in bone tissue was not detectable (Figure 2E). The blood vessels within the newly formed pulp tissue showed positive staining with CD31 (Figure 2F). Next, we tracked the transplanted GFP-labeled O9-1 cells. The GFP-positive cells were observed in the pulp-like tissue area (Figure 2G). Immunohistochemistry analysis revealed that the cells in the pulp-like tissue and odontoblast-like cells showed positive staining with GFP (Figure 2G). These results demonstrated that the transplanted O9-1 cells contribute to dentin–pulp complex regeneration.

### The Potential of iNCLCs for Vascularized Dentin–Pulp Complex Regeneration *in vivo*

**Figure 3:**
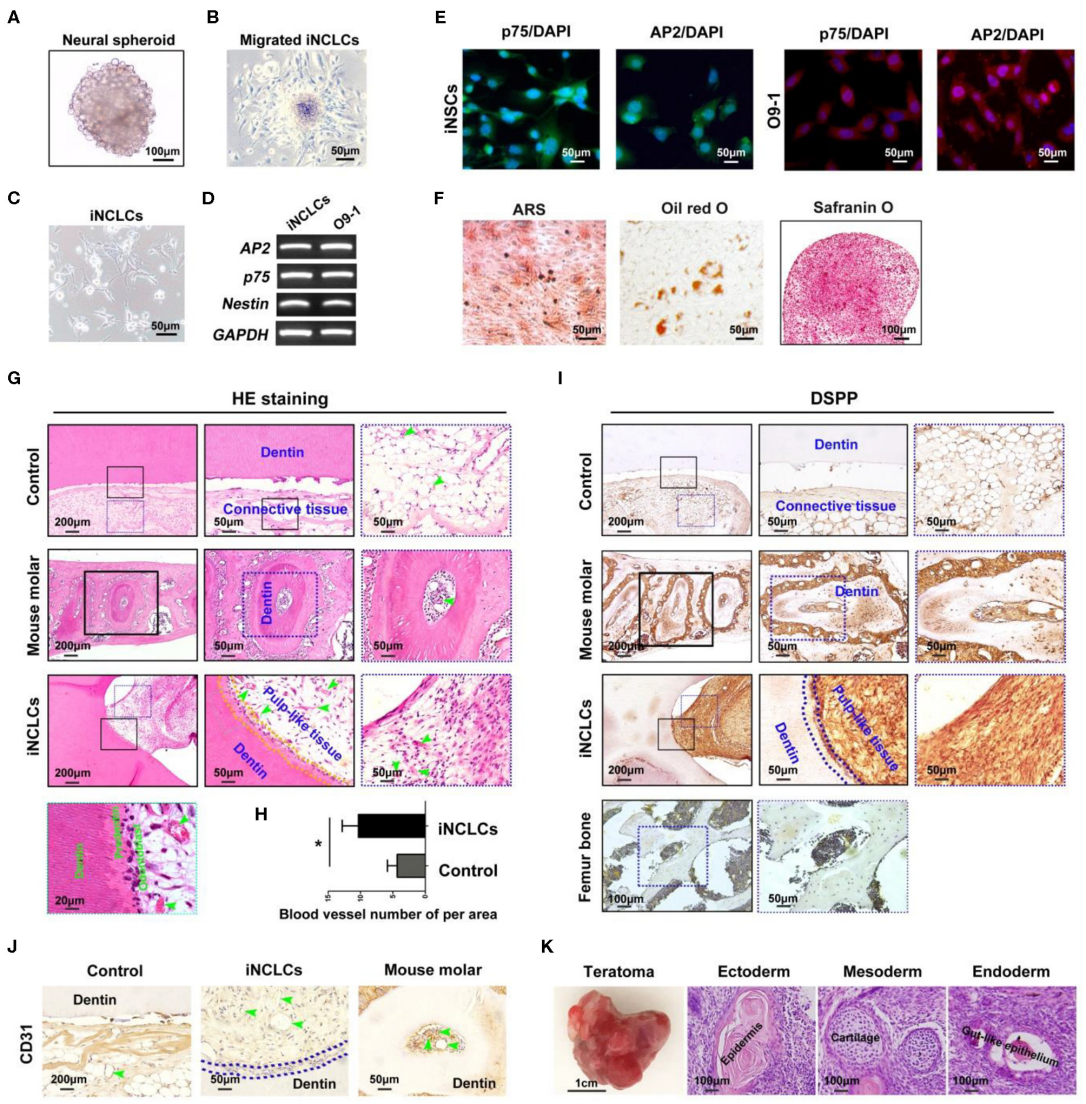
Neural crest-like cells derived from induced pluripotent stem cells (iPSCs) (iNCLCs) contribute to vascularized dentin–pulp complex regeneration *in vivo*. **(A)** iPSCs formed neural spheres under suspension culture. **(B)** The spheres attached to fibronectin-coated dish, and the cells migrated out from the spheres. **(C)** The morphology of the migrated cells after one time passage. **(D,E)** Reverse transcription PCR (RT-PCR) and immunocytochemistry were performed to evaluate the expression of neural crest-specific markers including p75 and AP2-α in iNCLCs. **(F)** Multilineage differentiation of iNCLCs. Osteogenic differentiation, positive staining with Alizarin Red S; adipogenic differentiation, positive staining with Oil Red O; chondrogenic differentiation, positive staining with Safranin O. **(G)** HE staining images of each group (the right two panels are the magnification of the boxed region; black arrowheads indicate odontoblast-like cells, yellow dash line area indicates newly formed dentin, and green arrowheads indicate blood vessel). **(H)** Histomorphometric analysis of newly formed vessel area; **(I)** Dentin sialophosphoprotein (DSPP) immunohistochemistry of each group (the right two panels are the magnification of the boxed region; dash blue line area indicates newly formed dentin); mouse femur bone tissue was treated as negative control group. **(J)** CD31 immunohistochemistry of each group (green arrowheads indicate blood vessels). **(K)** Transplanted tooth scaffold/iNCLCs complex formed teratoma, containing three germ-derived tissues including keratin-containing epidermal tissues (ectoderm), cartilage (mesoderm), and gut-like epithelial tissues (endoderm). *P < 0.05.

After suspension culture for 4 days, iPSCs formed neural spheres (Figure 3A). The neural spheres attached to the dishes, formed rosette-like structure, and gave rise to migratory cells with a stellate morphology (Figures 3B,C). The expression of neural crest-related markers including *AP2, p75*, and *Nestin* in iPSC-derived neural crest cells (iNCLCs) was similar to that of O9-1 cells (Figure 3D). Immunofluorescence analysis further revealed that iNCLCs, similar with O9-1 cells, showed positive staining with p-75 and AP2 (Figure 3E). The iNCLCs possessed multipotent differentiation potential and could differentiate into osteoblasts positive for mineral nodules based on Alizarin Red S staining, adipocytes positive for lipid droplets based on Oil Red O staining, and chondrocytes positive for Safranin O (Figure 3F). The flow cytometry study showed that ~80% of the iNCLCs were positive for p-75; the undifferentiated iPSCs were negative expression of p-75 (Figure S2).

The potential of iNCLCs for vascularized dentin-pulp regeneration was also investigate as shown in Figure 2A. Like O9-1 cells, the iNCLCs formed dentin-pulp complex within the tooth scaffold chamber (*n* = 3, total of 6 implants). Transplanted iNCLCs formed recognizable new dentin layers along the native dentin (Figure 3G). Odontoblast-like cells derived from iNCLCs constitute a single layer of cells on the surface of the pulp-like tissue below the newly formed dentin, which resemble with normal dentin-pulp tissue of the mouse molar (Figure 3G). High magnification images showed that odontoblast-like cells derived from both O9-1 cells and iNCLCs lined the surface of the newly formed predentin, ordered collagen fibers within predentin perpendicular to the odontoblast-like cell layer (Figures 2C, 3G). The pulp-like tissue showed rich blood vessels infiltration with intraluminal red blood cells (Figure 3G). The number of newly formed blood vessels within pulp-like tissue was significantly higher than that of the control group (Figure 3H). The pulp-like tissue and odontoblast-like cells derived from iNCLCs also showed positive staining with DSPP, similar with odontoblasts of the mouse molar. DSPP was not detectable or was almost negative in the mouse femur bone sample (Figure 3I). The blood vessels showed positive staining with CD31, which resemble with dental pulp tissue of the mouse molar (Figure 3J). One of the transplanted tooth scaffold/iNCLCs complex formed teratoma, containing three germ-derived tissues including keratin-containing epidermal tissues (ectoderm), cartilage (mesoderm), and gut-like epithelial tissues (endoderm) (Figure 3K).

### Odontoblastic Differentiation of O9-1 Cells and iNCLCs *in vitro*

**Figure 4:**
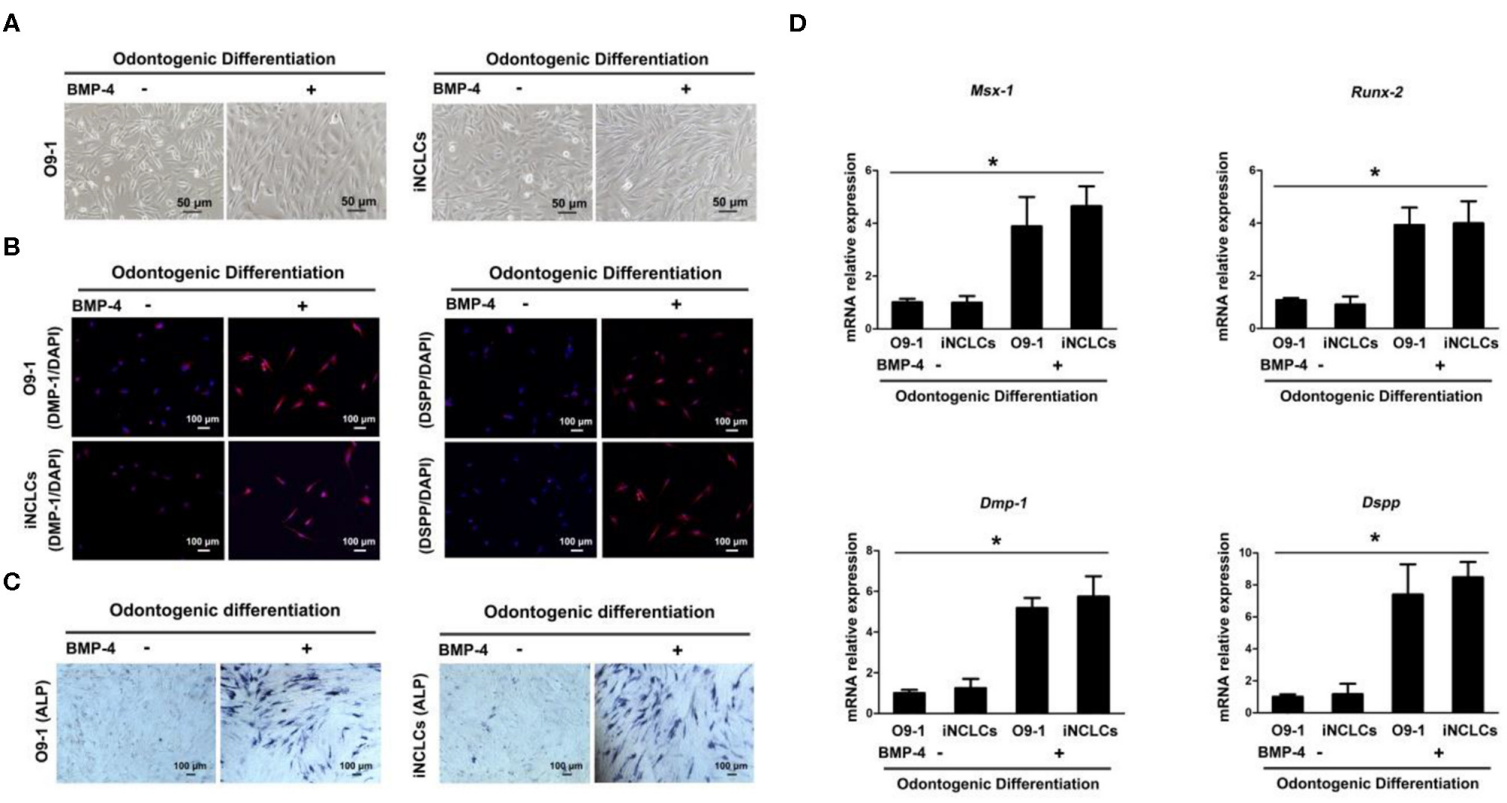
Odontoblastic differentiation of O9-1 cells and induced pluripotent stem cell-derived neural crest-like cells (iNCLCs) *in vitro*. **(A)** The morphology of differentiated O9-1 cells and iNCLCs under odontogenic differentiation with or without bone morphogenetic protein 4 (BMP-4) administration. **(B)** Immunofluorescence was performed to detect the expression of odontogenic markers dentin matrix acidic phosphoprotein 1 (DMP-1) and dentin sialophosphoprotein (DSPP). Nuclei were stained with 4′6-dia-mino-2-phenylindole (DAPI). **(C)** Alkaline phosphatase (ALP) staining. **(D)** The expression of *Msx-1, Runx-2, Dmp-1*, and *Dspp* was evaluated using real-time reverse transcription PCR (RT-PCR).

After 10 days of odontogenic induction culture, both O9-1 cells and iNCLCs revealed obvious morphological changes in the BMP-4 plus group compared with the BMP-4 minus group. The differentiated O9-1 cells and iNCLCs in BMP-4 plus group showed fibroblast-like spindle morphology (Figure 4A). The cells still remained with stellate morphology in the BMP-4 minus group (Figure 4A). Immunocytochemical analysis and ALP staining showed expression of DMP-1, DSPP, and ALP in the differentiated O9-1 cells and iNCLCs under the odontogenic induction with the administration of BMP-4 (Figures 4B,C). Without the stimulation of BMP-4, the cells showed negative staining with DMP-1, DSPP, and ALP (Figure 4C). The expression of odontogenic genes including *Msx-1, Runx-2, Dmp-1*, and *Dspp* was significantly upregulated in both O9-1 cells and iNCLCs BMP-4 plus group compared with that in both O9-1 and iNCLCs BMP-4 minus group (Figure 4D).

## Discussion

The cranial neural crest cells are crucial to craniofacial tissue and organ development including tooth organ. They give rise to various dental tissues including pulp, dentin, cement, and periodontal tissue (Chai et al., 2000). However, there were rare reports about the direct investigation of the odontogenic differentiation potential of neural crest cells within a developing tooth germ *in vitro*. During embryonic development, tooth is formed by sequential reciprocal interactions between dental epithelium and dental mesenchyme (Pispa and Thesleff, 2003; Tucker and Sharpe, 2004). Our results showed that GFP-labeled O9-1 cells differentiated into elongated odontoblasts adjacent to the inner epithelium within a developing tooth germ formed by epithelium and mesenchyme recombination. Moreover, GFP-labeled O9-1 cells regenerated dental-pulp-like tissue with a vascularized matrix resemble with the dentin-pulp tissue of the mouse molar when transplanted *in vivo* with tooth scaffold. Odontoblast-like cells derived from O-91 cells aligned the newly formed dentin surface, positive staining with odontoblast-specific marker DSPP. The odontoblast-like cells and the cells in pulp-like tissue were positive for GFP, which demonstrated that these cells were derived from O9-1 cells. These results provide direct evidence that neural crest cells are promising cell sources for the research of tooth development and dental tissue/tooth organ regeneration. However, neural crest is a temporary embryonic structure in vertebrates (Garcia-Castro et al., 2002). It is quite difficult to isolate adequate neural crest cells for stem-cell-based tooth development and regeneration research.

iPSCs, which can be generated from somatic cells by genetic manipulation, are invaluable experimental and therapeutic tools for the development of tissue regeneration technologies. Previous studies have demonstrated that iNCLCs (Seki et al., 2015) or primary ectomesenchymal stem cells (EMSCs) (Xing et al., 2016) could differentiate into odontoblast-like cells by gene transfection or through epithelial–mesenchymal interaction. However, these researches confirmed the odontogenic differentiation of iNCLCs or EMSCs only by detecting the expression of odontoblast-related marker such as DMP-1 and DSPP *in vitro*. There are no direct evidence to demonstrate that the odontoblast-like cells derived from iNCLCs are “functional odontoblasts,” which can contribute to dentin–pulp complex regeneration. In this study, we successfully directed iPSC differentiation into neural crest-like cells following the protocol established by a previous study (Otsu et al., 2012). iNCLCs are positive expression of the neural crest-related markers and possess multipotent differentiation ability. More than that, we further investigate the dental tissue regeneration potential of iNCLCs *in vivo*. Our result revealed that iNCLCs, similar with O9-1 cells, formed dentin–pulp complex when transplanted *in vivo* with tooth scaffold. Odontoblast-like cells derived from iNCLCs constitute a single layer of cells on the surface of the pulp-like tissue below the newly formed dentin, which resemble with normal dentin-pulp tissue of the mouse molar. To the best of our knowledge, this is the first to report that iNCLCs formed well-organized dentin-pulp tissue *in vivo*. Tumorigenicity is one of the major challenges hindering iPSC application (Lee et al., 2013; Neofytou et al., 2015). Notwithstanding that fact that part of iNCLCs/scaffold complex formed teratoma when transplanted *in vivo*, which may be caused by the undifferentiated iPSCs, cell sorting or longer induction time will be used for further study to avoid teratoma formation.

Multiple signaling molecules, including BMPs, FGFs, Shh, and Wnt proteins, have been implicated in mediating tooth development (Jussila and Thesleff, 2012). We further investigate the potential that induce NCCs to differentiate into odontogenic lineage *in vitro* via regulating the key signaling pathway involved in tooth development. At the initiate stage of tooth development, BMP-4 expresses in dental epithelium and then shifts to dental mesenchyme and induces dental mesenchymal cells differentiation (Vainio et al., 1993; Neubüser et al., 1997). Finally, the expression of BMP-4 is progressively restricted to the preodontoblastic cells underlying the enamel epithelium to regulate the differentiation of odontoblast and ameloblast. Our results revealed that BMP-4 induced dental mesenchymal-related marker *Msx1* and *Runx2* expression in both differentiated O9-1 cells and iNCLCs. Furthermore, the differentiated O9-1 cells and iNCLCs express odontoblastic-related markers including DMP-1 and DSPP. These results demonstrated that NCCs can be guided differentiation toward odontogenic lineage via regulating the crucial signaling of tooth development. Even though the detailed molecular mechanisms for inducing NCC differentiation toward to odontogenic lineage remain unknown and it is still a long way for whole tooth organ regeneration, these findings may be expected as the first step in the potential use of NCCs, especially unlimited iNCLCs, for tooth bioengineering.

## Conclusion

In this study, we demonstrated that NCCs can differentiate into DMP-1-positive odontoblasts within a developing tooth germ *in vitro*. Both O9-1 cells and iNCLCs formed well-organized vascularized dentin–pulp complex when transplanted *in vivo* with tooth scaffold. The O9-1 cells and iNCLCs also can differentiate into odontoblast-like cells *in vitro* by regulating BMP-4 signaling pathway. Taken together, NCCs, especially unlimited iNCLCs, are promising cell sources for the research related to tooth development and dental tissue/tooth organ regeneration.

## Data Availability Statement

The datasets generated for this study are available on request to the corresponding author.

## Ethics Statement

The studies involving human participants were reviewed and approved by Animal Research Committee of Ninth People's Hospital affiliated to Shanghai Jiao Tong University School of Medicine. The patients/participants provided their written informed consent to participate in this study. The animal study was reviewed and approved by Animal Research Committee of Ninth People's Hospital affiliated to Shanghai Jiao Tong University School of Medicine.

## Author Contributions

MZ, XZ, and MX contributed to conception and design, data acquisition, analysis, interpretation, and drafting of the manuscript. JL and RY contributed to data acquisition and drafting of the manuscript. KN, HE, and ZZ contributed to interpretation and critical revision of the manuscript. XJ contributed to conception and design, interpretation, and critical revision of the manuscript. All authors gave final approval and agreed to be accountable for all aspects of the work.

## Conflict of Interest

The authors declare that the research was conducted in the absence of any commercial or financial relationships that could be construed as a potential conflict of interest.
