# Supplementary material for: Investigate the Odontogenic Differentiation and Dentin–Pulp Tissue Regeneration Potential of Neural Crest Cells
Source: Front Bioeng Biotechnol. 2020 Jun 5;8:475. doi: 10.3389/fbioe.2020.00475 (PMC7290043; doi:10.3389/fbioe.2020.00475)
Supplement: Supplementary file 1 [file Table_1.pdf]

Supplementary Data

Table S1 Tooth formation success rate of mixed cell recombination

Origin	Percentage of different origin cells	Percentage of E14.5 dental mesenchyme cells	Number of recombination experiments	Tooth formation success number	Success rate %
hDPSCs	100%	0%	3	0	0
	50%	50%	6	0	0
	25%	75%	12	0	0
NIH3T3	100%	0%	3	0	0
	50%	50%	6	0	0
	25%	75%	6	2	33%
O9-1	100%	0%	3	0	0
	50%	50%	6	5	83%
	25%	75%	6	5	83%
