# Supplementary material for: Investigate the Odontogenic Differentiation and Dentin–Pulp Tissue Regeneration Potential of Neural Crest Cells
Source: Front Bioeng Biotechnol. 2020 Jun 5;8:475. doi: 10.3389/fbioe.2020.00475 (PMC7290043; doi:10.3389/fbioe.2020.00475)
Supplement: Supplementary file 2 [file Image_1.pdf]

Supplementary data

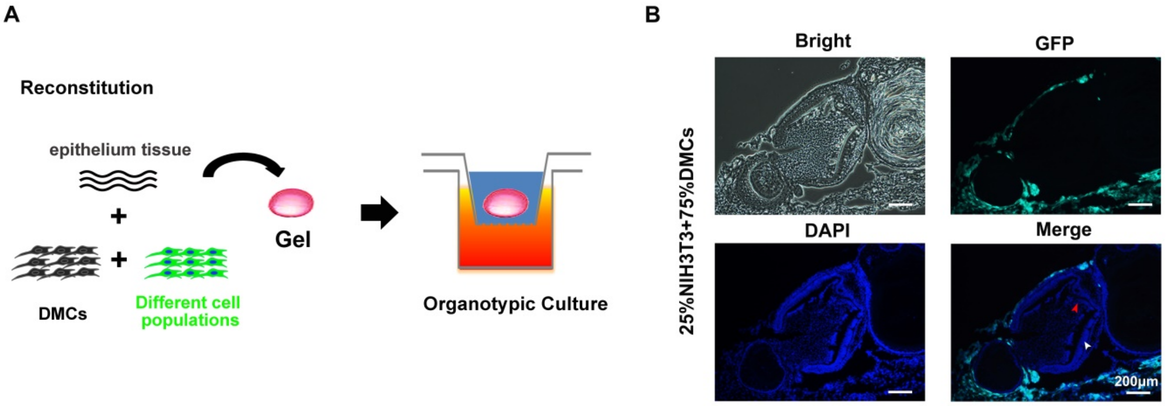

Figure S1. Schematic of tooth germ reconstitution and NIH3T3 cells failure to contribute to tooth germ formation. (A) The dental epithelial tissue and mesenchyme was isolated from molar tooth germ of ED 14.5 C57BL/6 mice. The dental mesenchyme was completely dissociated into single cells and mixed with GFP-labeled different cell population at different ratio as shown in Table S1. (B) No GFP^{+} (green) NIH3T3 cells could be observed inside the tooth primordia (red arrow indicates dental mesenchyme; white arrow indicates dental epithelium).
