# Supplementary material for: Investigate the Odontogenic Differentiation and Dentin–Pulp Tissue Regeneration Potential of Neural Crest Cells
Source: Front Bioeng Biotechnol. 2020 Jun 5;8:475. doi: 10.3389/fbioe.2020.00475 (PMC7290043; doi:10.3389/fbioe.2020.00475)
Supplement: Supplementary file 3 [file Image_2.pdf]

Supplementary data

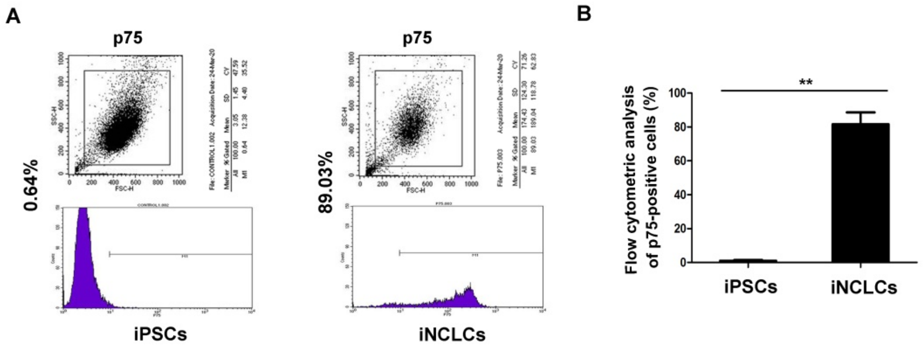

Figure S2. Representative flow cytometric analysis for p75-positve cells among undifferentiated iPSCs and iNCLCs.
